# The impact of bone graft type used to fill bone defects in patients undergoing ACL reconstruction with bone–patellar tendon–bone (BPTB) autograft on kneeling, anterior knee pain and knee functional outcomes

**DOI:** 10.1007/s00590-023-03624-9

**Published:** 2023-07-01

**Authors:** Ali Fares, Alexandre Hardy, Yoann Bohu, Alain Meyer, Karam Karam, Nicolas Lefevre

**Affiliations:** Chirurgie du Sport, Clinique du Sport Paris V, Ramsay-Générale de Santé, Paris, France

**Keywords:** BTPT graft, ACL, Glassbone, Collapat, Osteopure, Kneeling pain

## Abstract

**Purpose:**

Multiple different materials are used for filling bone defects following bone–patellar tendon–bone (BPTB) graft ACL reconstruction surgery. The theoretical objective being to minimize kneeling pain, improve clinical outcomes and reduce anterior knee pain following surgery. The impact of these materials is assessed in this study.

**Methods:**

A prospective monocentric cohort study was conducted from January 2018 to March 2020. There were 128 skeletally mature athletic patients who underwent ACL reconstruction using the same arthroscopic-assisted BPTB technique, with a minimum follow-up of two years identified in our database. After obtaining approval from the local ethics committee, 102 patients were included in the study. Patients were divided into three groups based on type of bone substitute. The Bioactive glass 45S5 ceramic Glassbone™ (GB), collagen and hydroxyapatite bone void filler in sponge form Collapat® II (CP), and treated human bone graft Osteopure®(OP) bone substitutes were used according to availability. Clinical evaluation of patients at follow-up was performed using the WebSurvey software. A questionnaire completed in the 2nd post-operative year included three items: The ability to kneel, the presence of donor site pain, and the palpation of a defect. Another assessment tool included the IKDC subjective score and Lysholm score. These two tools were completed by patients preoperatively, and postoperatively on three occasions (6 months, 1 year, and 2 years).

**Results:**

A total of 102 patients were included in this study. In terms of Kneeling pain, the percentage of GB and CP patients’ who kneel with ease were much higher than that of OP patients (77.78%, 76.5% vs 65.6%, respectively). All three groups experienced an important increase in IKDC and Lysholm scores. There was no difference in anterior knee pain between the groups.

**Conclusion:**

The use of Glassbone® and Collapat II® bone substitutes reduced the incidence of kneeling pain compared to Osteopure®. There was no influence of the bone substitute type on the functional outcome of the knee or on the anterior knee pain at two years of follow.

## Introduction

Anterior cruciate ligament (ACL) injuries are among the most common knee injuries, and ACL reconstruction (ACLR) is a widely performed operation [1, 2]. The bone–patellar tendon–bone (BPTB) and hamstring tendon autografts are two of the most commonly used autografts for ACLR [3, 4]. Furthermore, BPTB autograft has long been the gold standard for treatment, as its bone blocks at both ends of the graft provide high fixation strength [5, 6]. Nevertheless, 15–60% of patients may complain of long-term post-operative anterior knee pain during daily living or physical activities. Kneeling pain and donor site defects are also frequently observed [7–12].

It has been argued that patellar and tibial bone defects following graft harvesting are a risk factor impacting anterior knee pain in BPTB patients. Other claims are that infrapatellar nerve damage during graft harvesting is responsible for this morbidity [13, 14]. Recently, a systematic review showed that BTBP ACLR patients, whose bone defects were filled, have fewer post-operative knee complaints and better knee functional outcomes than patients treated without defect filling [8]. The most common bone grafts used are either autologous bone grafts, allogeneic bone grafts or synthetic substitutes [15–17]. Nonetheless, no study has compared the outcomes of different types of bone graft in terms of kneeling and functional outcomes in BTBP ACLR patients.

Such bone grafting options include the Bioactive glass 45S5 ceramic, such as Glassbone® (GB); collagen and hydroxyapatite bone void filler in sponge form, such as Collapat II® (CP), and treated human bone graft, such as Osteopure® (OP).

This cohort study aimed to investigate the influence of these bone graft types on kneeling and knee functional outcomes. The hypothesis was that there was no superiority of one substitute over another.

## Materials and methods

**Fig. 1:**
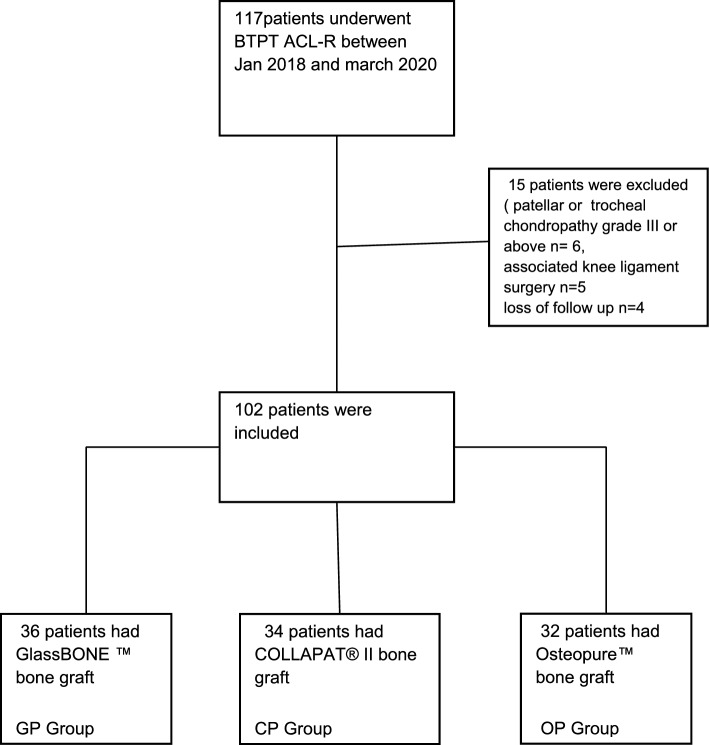
Flowchart showing the distribution of patients ﻿into three groups

A prospective single-center cohort study of the French prospective ACL Study [FAST] (NCT02511158) was performed, including all patients who performed ACLR using BPTB autograft between 2018 and 2020 by 4 senior surgeons. The study was approved by the local ethics committee (Comité de Protection des Personnes IDF VI), and informed consent was obtained from all patients. A retrospective analysis of the prospectively filled data, with a minimum follow-up of two years was performed. One hundred and two patients undergoing ACL reconstruction using BPTB autograft were assessed. Clinical evaluation of patients at follow-up was performed by the surgeons and data was entered in the WebSurvey software. The inclusion criteria were ACL reconstruction using the BTBP technique, athletes, a minimum of two years of post-operative follow-up and an age over 18 years. Exclusion criteria were associated knee ligament injury requiring surgical treatment, chondropathy of grade III or higher involving the trochlea or the patella, immune rheumatologic pathologies, preexisting anterior knee pain, and prior surgery on the same knee. Patients were divided into three groups according to bone substitute type. Three different bone substitutes were used according to availability at the time of surgery: Glassbone™, Collapat® II, and Osteopure®. The timeline is detailed in Fig. 1.

### Bone substitutes

Osteopure® is a bone allograft harvested from a resected live human femoral head, and treated by sterilization at 25 kGy.

**Fig. 2:**
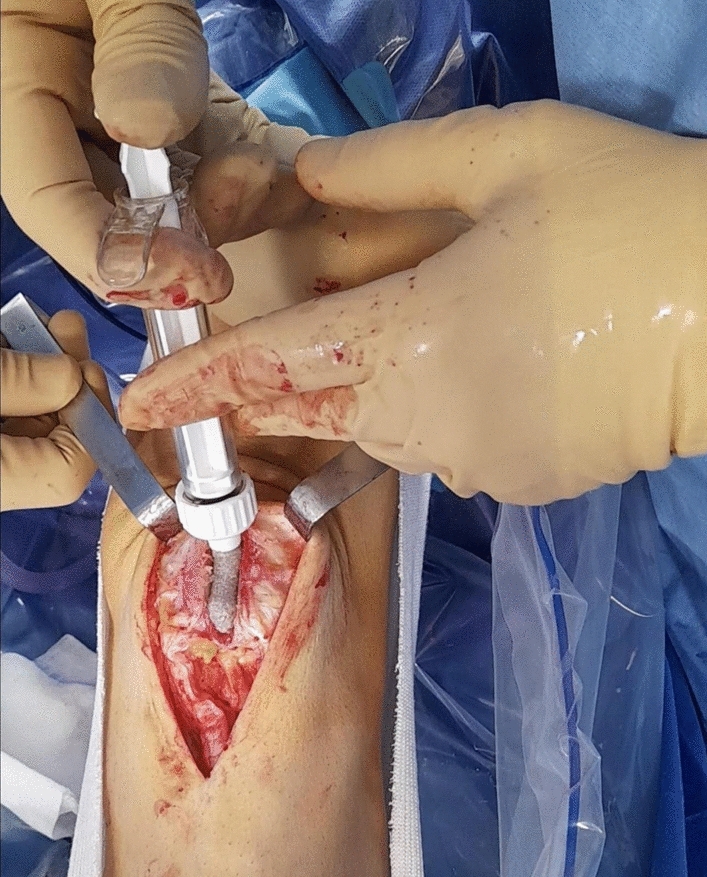
Intra-operative photograph showing the patellar defect being filled with Glassbone allograft

Glassbone® is a bioactive glass which is 100% synthetic, biocompatible, and osteoconductive and can integrate with the bone and soft tissue as a defect filler (Fig. 2). It is composed of a mixture of oxides (45% SiO_2_, 24.5% CaO, 25.5%, Na_2_O, and 6% P_2_O_5_ in weight %) [18–25].

Collapat® II is a bone void filler presented in spongy form. It is composed of a collagen structure in which hydroxyapatite granules are dispersed. The granules of hydroxyapatite give the material its osteoconductive properties [23].

### Patient follow-up and data collection

Three tools were used for data collection at various time points. First, a questionnaire assessed the international knee documentation committee (IKDC) [24] subjective score and Lysholm score [25]. These two tools were completed by patients at four time points: first pre-operatively, and at 6 months, one year and two years postoperatively. Another standardized questionnaire was sent by email to the participants 4 months postoperatively. This was repeated at the 6 months, one year and two years marks following surgery. This questionnaire was made available online via a link to the WebSurvey software (websurvey.fr). If patients failed to answer, a first reminder was made via email, and a second by telephone call. Finally, a questionnaire was sent at the second post-operative year. It evaluated 3 items: The ability to kneel assessed by the subsection of Hacken’s questionnaire [26], the presence of donor site pain during sports or daily activities assessed by the Numerical Rating Scales (NRS) [27], and the sensation of a defect at the donor site. No formal sample size calculation was done. All eligible patients who underwent ACLR BPTB graft between 2018 and 2020 at our institution were included in the study.

### Surgical protocol

Under spinal anesthesia, BPTB autografts were used to reconstruct the ACL. A 9 cm para median incision was performed, the paratenon was dissected carefully, and the a middle third of the patellar tendon was harvested with approximately 10 × 10 × 20 mm bone blocks from the patella and tibia. The ACL remnant was preserved. The tibial bone tunnel was prepared to be 10 mm in diameter. The tibial tunnel was created with a specific guide (*Acufex; Smith & Nephew*). The femoral tunnel was 10 mm in diameter and placed at the origin of the native ACL, on the medial surface of the lateral femoral condyle using an inside-out technique. The BTB autograft was fixed in the femoral tunnel with a non-absorbable interference screw (*Softsilk; Smith & Nephew*) or absorbable pins using the RigidFix system (*DePuy Synthes, Mitek rigid fix*), depending on surgeon preference. After tensioning the graft, the patellar bone block was stabilized in the tibial tunnel with another interference bioabsorbable screw (*Smith & Nephew*). Finally, the bony defects were filled with the corresponding bone graft and the paratenon was sutured over the bone substitutes.

### Post-operative rehabilitation protocol

All patients underwent the same rehabilitation protocol. Immediate full weight-bearing with an articulated brace was allowed using crutches for the first 3 weeks to avoid unexpected falls. Physiotherapy for analgesia, patella mobilization, progressive full range-of-motion exercises, and isometric quadriceps contraction exercises were allowed, with the expectation that at one-month postoperatively, the patient would have a normal gait, full extension and 110° of flexion. In the case of meniscal suture, knee flexion while weight-bearing was limited to 120° for two months postoperatively.

### Statistical analysis

All statistical analyses were performed using the IBM SPSS statistics software. Categorical variables were summarized as frequencies and percentages. Continuous variables were presented as means, standard deviations and ranges. One-way ANOVA was used to compare the mean IKDC and Lysholm scores, as well as the change in these scores between the three groups. Repeated measures ANOVA was used to compare the IKDC and Lysholm scores at different time points within each group. Pearson’s Chi-square test or Fisher exact test were used to assess the association of gender, ability to kneel, and internal pain between the three groups. All tests were two-sided and a *p* < 0.05 was considered statistically significant.

## Results

One hundred and seventeen patients underwent ACLR using BPTB autograft. Of those, 102 (87.17%) were included in this study, and 15 (12.83%) were excluded. Among the 102 patients, 36 (35.29%) patients were placed in the Glassbone® group (group 1), 34 (33.33%) in the Collapat II® group (group 2), and the remaining 32 (31.37%) in the Osteopure® group (group 3). The three groups had no significant differences in terms of age (*p* = 0.127) and gender (*p* = 0.511). The mean age was 32.17 ± 8.20 years. Men represented 78.43% of the studied population. Detailed demographic characteristics are described in Table 1.

**Table 1:** Patients demographic characteristics

		Overall (*n* = 102)	GlassBone Group (*n* = 36)	Collapat II Group (*n* = 34)	Osteobank Group (*n* = 32)	*P* value
Age (years)	Mean ± SD (range)	32.17 ± 8.20 (18–56)	30.36 ± 8.38 (18–48)	34.32 ± 7.57 (21–54)	31.91 ± 8.36 (20–56)	0.127*
Gender *n* (%)	Male	80 (78.43)	30 (83.33)	27 (79.41)	23 (71.88)	0.511^‡^
	Female	22 (21.57)	6 (16.67)	7 (20.59)	9 (28.13)	

SD: Standard deviation. *p value was calculated using one-way ANOVA. ^‡^*p* value was calculated using Chi-square test or Fisher exact test. *P* < 0.05 was considered as statistically significant

Among the 102 patients studied, 27 (26.47%) complained of Kneeling pain. There was a significant difference between the three groups (*p* = 0.045), the percentage of Glassbone and Collapat patients’ who kneel comfortably was significantly higher than that of osteobank patients (77.78%, 76,5% vs 65.6%, respectively). Moreover, the percentage of osteobank patients who were unable to kneel on hard surfaces was higher than that of Glassbone and Collapat patients (8% vs 2,78; 2.94%, respectively).

In the study population, 31 (30.39%) patients had anterior knee pain with an average of 3.77 ± 1.50 on the NRS scale. The percentage of patients experiencing anterior knee pain was 30.56% (mean 3.64 ± 1.03), 29.41% (mean 3.80 ± 1.69), and 31.25% (mean 3.90 ± 1.85) in groups 1, 2 and 3, respectively (*p* value 0.987).

The clinical characteristics are described in Table 2.

**Table 2:** Patient clinical characteristics

		Overall (*n* = 102)	GlassBone group (*n* = 36)	Collapat II group (*n* = 34)	Osteobank group (*n* = 32)	*P* value
Kneeling *n* (%)	No pain with kneeling	75 (73.53)	28 (77.78)	26 (76.5)	21 (65.63)	0.045^‡^
	Mild pain with kneeling	13 (12.75)	6 (16.66)	5 (14.71)	2 (6.25)	
	Inability to kneel on hard surfaces	10 (9.80)	1 (2.78)	1 (2.94)	8 (25.0)	
	Completely, unable to kneel	4 (3.92)	1 (2.78)	2 (5.88)	1 (3.12)	
Anterior knee pain *n* (%)	No	71 (69.61)	25 (69.44)	24 (70.59)	22 (68.75)	0.987^‡^
	Yes	31 (30.39)	11 (30.56)	10 (29.41)	10 (31.25)	
If yes, NRS score (*n* = 31)	Mean ± SD (range)	3.77 ± 1.50 (1–7)	3.64 ± 1.03 (2–5)	3.80 ± 1.69 (2–7)	3.90 ± 1.85 (1–6)	0.925*
Defect sensation	Yes No	0 102 (100%)	0 36 (100%)	0 34 (100%)	0 32 (100%)	NA

SD: Standard deviation. *p value was calculated using one-way ANOVA. ^‡^*p* value was calculated using Chi-square test or Fisher exact test. *P* < 0.05 was considered as statistically significant

The IKDC score was significantly improved in the three groups compared to the pre-operative status (*P* < 0.01), as detailed in Table 3.

**Table 3:** IKDC score at each time point by type of the bone substitute groups

	Overall (*n* = 102)	GlassBone group (*n* = 36)	Collapat II group (*n* = 34)	Osteobank group (*n* = 32)	*P* value*
Pre-operative	56.94 ± 16.11 (13–90)	56.67 ± 14.43 (26–84)	60.35 ± 15.28 (32–90)	53.63 ± 18.38 (13–84)	0.238
6 months post-op	67.45 ± 13.37 (20–95)	69.22 ± 9.54 (36–86)	66.65 ± 14.14 (20–83)	66.31 ± 16.15 (33–95)	0.615
1 year post-op	75.18 ± 14.07 (26–99)	76.42 ± 9.26 (54–89)	74.82 ± 16.58 (26–99)	74.16 ± 15.89 (39–98)	0.794
2 years post-op	80.08 ± 14.54 (26–100)	81.17 ± 10.61 (55–97)	81.18 ± 15.97 (26–100)	77.69 ± 16.79 (40–98)	0.537
Pre-op to 2-year post-op change	23.14 ± 16.99 (− 15–73)	24.50 ± 15.64 ((− 4)–60)	20.52 ± 15.55 ((− 8)–55)	24.06 ± 19.94 ((− 15.0)–73)	0.624
*P* value^†^		< 0.001			

SD: Standard Deviation; IKDC: International Knee Documentation Committee. Data were expressed as mean ± SD (range). *P value was calculated using one-way ANOVA. ^†^*P* value was calculated using repeated measure ANOVA.* P* < 0.05 was considered as statistically significant

In group 1, the mean IKDC score increased from 56.67 ± 14.43 (range 26–84) pre-operatively to 69.22 ± 9.54 (range 36–86), 76.42 ± 9.26 (range 54–89) and 81.17 ± 10.61 (range 55–97) at 6 months, 1 year and 2 years postoperatively respectively, with a statistically significant mean change of 24.50 ± 15.64 (range (− 4)–60) (*p* < 0.001).

In group 2, the mean IKDC score increased from 60.35 ± 15.28 (range 32–90) at pre-operative to 66.65 ± 14.14 (range 20–83), 74.82 ± 16.58 (range 26–99) and 81.18 ± 15.97 (range 26–100) at 6 months, 1 year and 2 years post-operative respectively, with a statistically significant mean change of 20.52 ± 15.55 (range (− 8)–55) (*p* < 0.001).

In group 3, the mean IKDC score increased from 53.63 ± 18.38 (range 13–84) at pre-operative to 66.31 ± 16.15 (range 33–95), 74.16 ± 15.89 (range 39–98) and 77.69 ± 16.79 (range 40–98) at 6 months, 1 year and 2 years post-operative respectively, with a statistically significant mean change of 24.06 ± 19.94 (range (– 15.0)–73) (*p* < 0.001).

There was no statistically significant difference in the mean IKDC score between the three groups (*p* > 0.05).

**Fig. 3:**
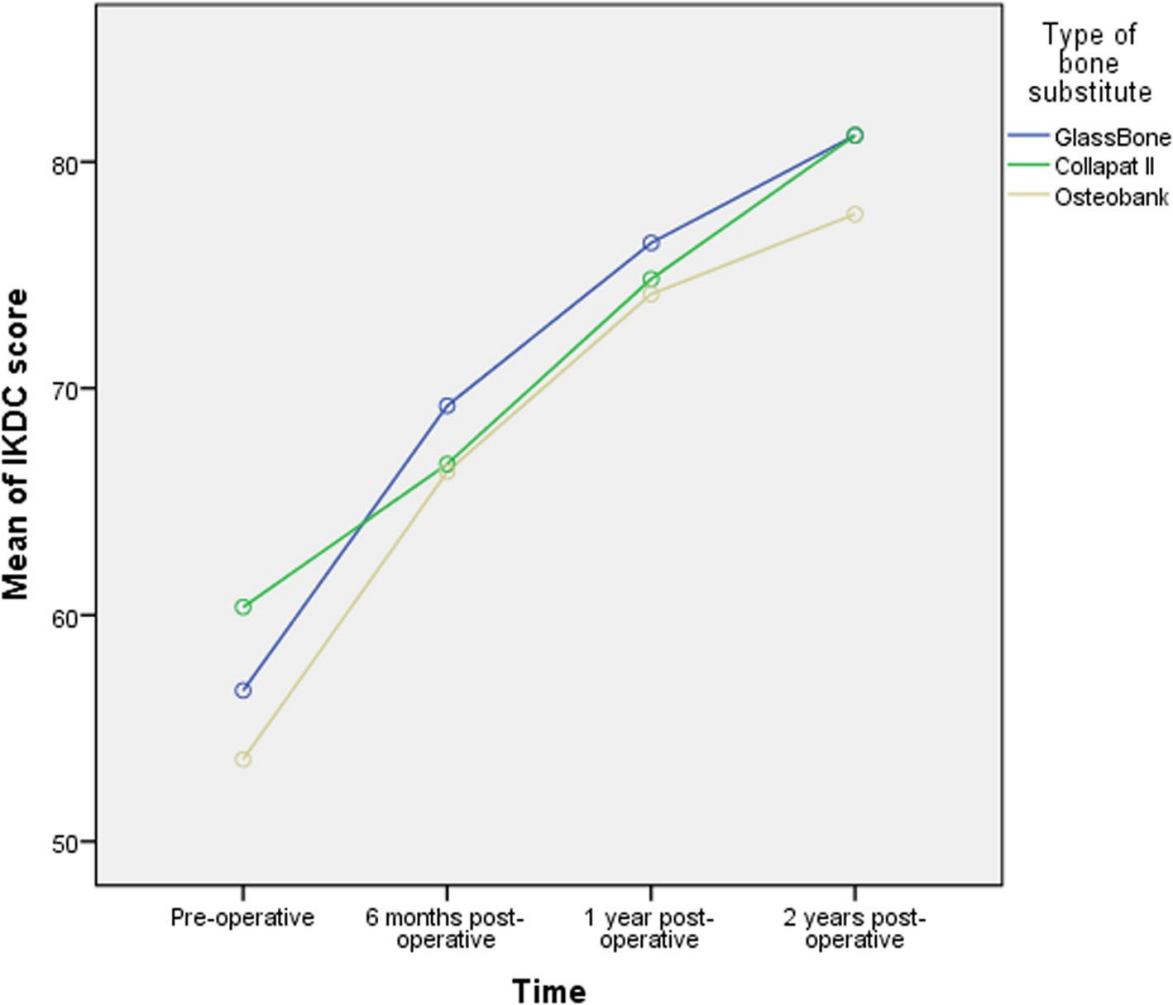
Evolution of the IKDC score over time

The evolution of IKDC score by time in the three groups is shown in Fig. 3.

The Lysholm score was significantly improved in the three groups compared to the pre-operative status (*p* < 0.01) as detailed in Table 4.

**Table 4:** Lysholm score at each time point by type of the bone substitute groups

	Overall (*n* = 102)	GlassBone group (*n* = 36)	Collapat II group (*n* = 34)	Osteobank group (*n* = 32)	*P* value*
Pre-operative	65.55 ± 18.09 (2–99)	67.53 ± 15.18 (28–95)	67.88 ± 18.06 (15–95)	60.84 ± 20.61 (2–99)	0.207
6 months postoperative	79.72 ± 13.72 (22–100)	81.33 ± 11.26 (44–95)	81.41 ± 16.02 (22–99)	76.09 ± 13.32 (49–100)	0.198
1 year postoperative	84.44 ± 12.02 (31–100)	86.53 ± 10.24 (60–99)	85.68 ± 13.43 (31–100)	80.78 ± 11.82 (56–98)	0.110
2 years postoperative	87.46 ± 12.11 (30–100)	89.78 ± 9.90 (52–100)	87.18 ± 13.78 (30–100)	85.16 ± 12.37 (56–100)	0.290
Pre-operative to 2-year change	21.91 ± 17.26 (− 11–80)	22.25 ± 15.21 ((− 6)–66)	19.29 ± 14.18 ((− 1)–80.0)	24.31 ± 21.93 ((− 11)–80)	0.497
*P* value†		< 0.001			

Data were expressed as mean ± SD (range). *p value was calculated using one-way ANOVA. ^†^*p* value was calculated using repeated measure ANOVA. *P* < 0.05 was considered as statistically significant

In group 1, the mean Lysholm score increased from 67.53 ± 15.18 (range 28–95) at pre-operative to 81.33 ± 11.26 (range 44–95), 86.53 ± 10.24 (range 60–99) and 89.78 ± 9.90 (range 52–100) at 6 months, 1 year and 2 years post-operative respectively, with a statistically significant mean change of 22.25 ± 15.21 (range (− 6)–66) (*p* < 0.001).

In group 2, the mean Lysholm score increased from 67.88 ± 18.06 (range 15–95) at pre-operative to 81.41 ± 16.02 (range 22–99), 85.68 ± 13.43 (31–100) and 87.18 ± 13.78 (range 30–100) at 6 months, 1 year and 2 years post-operative respectively, with a statistically significant mean change of 19.29 ± 14.18 (range (− 1)–80.0) (*p* < 0.001).

In group 3, the mean Lysholm score increased from 60.84 ± 20.61 (range 2–99) at pre-operative to 76.09 ± 13.32 (range 49–100), 80.78 ± 11.82 (range 56–98) and 85.16 ± 12.37 (range 56–100) at 6 months, 1 year and 2 years post-operative respectively, with a statistically significant mean change of 24.31 ± 21.93 (range (− 11)–80) (*p* < 0.001).

Similarly, to the IKDC score, no statistically significant difference in the mean Lysholm score between the three groups was detected (*p* > 0.05).

**Fig. 4:**
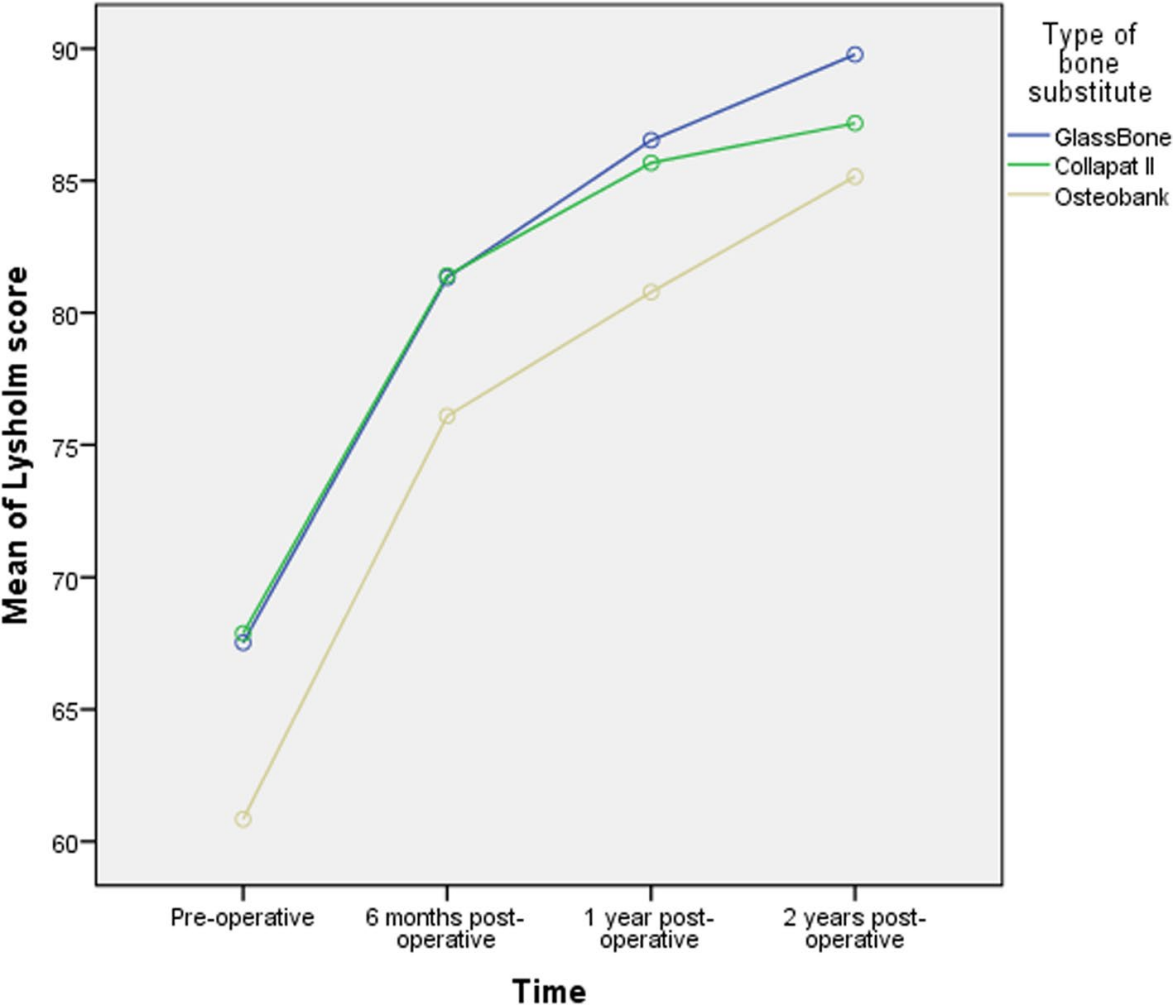
Evolution of the Lysholm score over time

The evolution of Lysholm score by time in the three groups is shown in Fig. 4.

All patients in the study, having subjectively assessed their knees, found no sensation of a bony defect at 2 years follow-up (Table 2). One incidence of a superficial abscess at the surgical site was observed in group 2. In this patient, the substitute was excized and an extra-articular debridement was needed to manage the complication.

## Discussion

This study was designed to compare outcomes of ACL reconstruction with a BPTB autograft using one of three bone substitutes to fill the harvested zone. The primary finding was that the patients who received synthetic bone grafts, (Glassbone or Collapat II) had a higher percentage of painless kneeling compared to those who had Osteopure allograft filling. However, there was no significant difference between the three groups in terms of IKDC, Lysholm, and anterior knee pain.

Kneeling pain was evaluated using one item of Hacken’s questionnaire [26]. The higher incidence of kneeling pain of patients of group 3 compared to patients from other groups might be due to the persistent inflammatory response or suboptimal bone consolidation caused by the Osteopure allograft [28]. The incidence of painless kneeling in this study was 73.53% overall, with 77.78% of the Glassbone patients reporting no pain. After reviewing the literature, it was found that this was higher than the study by Taylor et al. (62%) [29] and lower than the study by Hacken et al. (90.4%) [26]. In both of those studies, cancellous autograft had been used for filling the bone defects. On the other hand Barrenius [30], Leitge [31] and Liden [32] who did not fill the bone defects registered a higher incidence of kneeling pain than the findings of the present study.

From a cosmetic standpoint, filling the defect with allograft improves appearance, a common patient concern, and abolishes the sensation of a bone gap or defect at the donor site. This allows avoidance of a further patient complaint during follow-up visits [33].

A major concern with using BPTB autograft for ACLR is donor site morbidity, specifically anterior knee pain [26]. Surgeons have attempted to change the harvesting technique in order to decrease this complication [12, 34], others have elected to change the graft type, like Schande et al. who used serum albumin-coated bone allograft [35]. Cervelline et al. filled the donor sites with PRP gel [36]. Nelson et al. also described a new technique for filling the defect [37]. Naresh et al. elected to fill the defects with ceramic bone graft but the results were non-satisfactory [38]. Our study aimed to identify the influence of different types of bone substitutes on anterior knee pain and found similar results in all three groups. The results are comparable to the findings of a systematic review by Lameire et al. who showed that filling defects decreased anterior knee pain, kneeling pain and extension loss [8].

No study evaluated and compared the functional outcome and donor site morbidity between Glassbone, Collapat II, or Osteopure bone substitutes in the BPTB ACLR population. Although there are numerous scoring tools to quantify ACLR patients’ results [39], IKDC and Lysholm scores were chosen for this study, as they are standard instruments for evaluating patients postoperatively and two of the most commonly reported functional outcome scores [8, 26, 40]. Both scores in the present study showed satisfactory recovery in all three groups without significant difference between groups. Subjective IKDC ranged from 77 to 81 after two years following ACLR, and the Lysholm score ranged from 85 to 90. Comparing our results to the systematic review by Lameire et al., it is observed that the IKDC scores are similar, but the Lysholm scores are inferior [8]. Overall, however, it was determined that the type of bone substitute did not affect the functional knee outcome.

There was no complication reported in terms of wound healing except for a patient from group 2 who exhibited an extrusion of part of the bone substitute and needed surgical intervention. This case might be a coincidence, and conclusions cannot be drawn based on a single exceptional case. It is important to mention that this is the first study that showed the tolerance of donor sites to Glassbone in BPTB ACLR patients. There were no complications detected which might be due to its bacteriostatic activity [22]. No patellar fracture occurred in any patient of the three groups. This is similar to the results of Alexander et al. [41].

This study shows that the kneeling pain was higher in Osteopure group. We can only speculate about this discrepancy. Osteopure is a natural bone block which needs to be cut into shape to fill a defect. As a rigid substitute, it is more difficult to fully fill the defect with it compared to the other softer substitutes (Glassbone and Collapat). Furthermore, it is composed of cancellous bone. Perhaps the replacement of cortical bone from the patella and tibia with spongy bone from the bone substitute affects rigidity and therefore leads to more pain in this patient population. Bone graft healing is a sequential process involving inflammation revascularization, osteogenesis remodeling, and incorporation into the host skeleton to form a mechanically efficient structure so this process might be different between the three allograft types. Further studies would be needed to possibly give a more accurate response in the future.

The strengths of this study were the high response rate, the 2-year follow-up period and the prospective administration of questionnaires.

There were, however, several limitations. First, this was not a randomized trial, and it was not a blinded study. Although patients may have been blinded, the surgeons would not have been. Furthermore, the bone substitute used was done so based on availability, rather than random assignment. Secondly, although the operations were all performed in the same institution, different surgeons participated in the study and performed surgery. Moreover, concomitant meniscus injury was not part of the exclusion criteria. This likely affects standardization of the procedure and may cause variability in patient outcomes.

## Conclusion

This study finds that there is a reduced incidence of kneeling pain and discomfort when using bone substitutes such as Glassbone® and Collapat II® compared to allografts such as Osteopure® at a 2-year follow-up. However, the choice of bone-filling material influences neither functional knee outcomes, nor anterior knee pain at 2 years postoperatively.
